# Fabrication of Bi_2_MoO_6_ Nanosheets/TiO_2_ Nanorod Arrays Heterostructures for Enhanced Photocatalytic Performance under Visible-Light Irradiation

**DOI:** 10.3390/nano12030574

**Published:** 2022-02-08

**Authors:** Di Zhou, Rui Du, Zhenglong Hu, Shu Gao, Yafang Tu, Yunfei Fu, Guang Zheng, Youhua Zhou

**Affiliations:** 1 School of Optoelectronic Materials and Technologies, Jianghan University, Wuhan 430056, China; dizhou@jhun.edu.cn (D.Z.); durui199602@163.com (R.D.); milton_gao@jhun.edu.cn (S.G.); yafangtu@163.com (Y.T.); yffu89@jhun.edu.cn (Y.F.); xinge@whut.edu.cn (Y.Z.); 2 Laboratory of Low-Dimension Functional Nanostructures and Devices, Hubei University of Science and Technology, Xianning 437100, China

**Keywords:** Bi_2_MoO_6_, TiO_2_ nanorod arrays, heterostructure, photodegradation, solvothermal method

## Abstract

Bi_2_MoO_6_/TiO_2_ heterostructures (HSs) were synthesized in the present study by growing Bi_2_MoO_6_ nanosheets on vertically aligned TiO_2_ nanorod arrays using a two-step solvothermal method. Their morphology and structure were characterized by scanning electron microscopy (SEM) and X-ray diffraction (XRD), respectively. Excellent visible-light absorption was observed by UV–Vis absorption spectroscopy, which was attributed to the presence of the Bi_2_MoO_6_ nanosheets with a narrow-band-gap. The specific surface area and pore volume of the photocatalysts were significantly increased due to the hierarchical structure composed of Bi_2_MoO_6_ nanosheets and TiO_2_ nanorods. The photoluminescence and photoelectrochemical characterizations showed improved separation and collection efficiency of the Bi_2_MoO_6_/TiO_2_ HSs towards the interface charge carrier. The photocatalytic analysis of the Bi_2_MoO_6_/TiO_2_ HSs demonstrated a significantly better methylene blue (MB) degradation efficiency of 95% within 3 h than pristine TiO_2_ nanorod arrays under visible-light irradiation. After three photocatalytic cycles, the degradation rate remained at ~90%. The improved performance of the Bi_2_MoO_6_/TiO_2_ HSs was attributed to the synergy among the extended absorption of visible light; the large, specific surface area of the hierarchical structure; and the enhanced separation efficiency of the photogenerated electron-hole pairs. Finally, we also established the Bi_2_MoO_6_/TiO_2_ HSs band structure and described the photocatalytic dye degradation mechanism. The related electrochemical analysis and free-radical trapping experiments indicated that h^+^, ·O_2_^−^ and ·OH have significant effects on the degradation process.

## 1. Introduction

Light- and catalysis-assisted removal of pollutants and water electrolysis are popular processes because of their environmental friendliness. Titanium dioxide (TiO_2_) is a popular photocatalyst because of its favorable electron mobility, resistance to photocorrosion, low cost, and low toxicity [1,2,3,4]. Nevertheless, its poor solar-light utilization, due to its band-gap value (3.0–3.2 eV) and high photo-charge-carrier recombination rate [5,6], limit the extensive application of anatase and rutile. These drawbacks were somewhat successfully overcome by TiO_2_ dye-sensitization, doping, and coupling with other metals and their oxides [7,8,9,10]. The most promising method is the combination of TiO_2_ with materials possessing narrow band-gaps. The resulting heterostructures (HSs) demonstrate an extended (to the visible-light spectrum) optical absorption and increased separation of charge carriers [11,12,13,14].

Bismuth (III)-containing oxides, such as Bi_2_O_3_, BiOI, BiOCl, BiVO_4_, Bi_4_Ti_3_O_12_, and Bi_2_WO_6_, have attracted much interest due to their superior photocatalytic and physicochemical performances [15,16,17,18]. Importantly, Bi_2_MoO_6_ is a layered oxide with an Aurivillius structure that possesses visible-light-driven photocatalytic activity for water and organic electrolysis and decomposition [19,20]. Interestingly, some studies reported that coupling Bi_2_MoO_6_ with TiO_2_ yielded HSs with enhanced photocatalytic performance. For instance, the flake-like Bi_2_MoO_6_ grown on TiO_2_ films demonstrated excellent significant visible-light self-cleaning properties [21], which were attributed to the synergy between the individual components of these HSs, including superhydrophilicity and effective charge-carrier separation [21]. Pan et al. [22] reported that Bi_2_MoO_6_/TiO_2_ HS microspheres exhibited excellent photocatalytic activity towards phenol and nitrobenzene decomposition under visible light. Zhang et al. [23] prepared Bi_2_MoO_6_/TiO_2_ HSs with two different morphologies using Bi_2_MoO_6_ nanoparticles and nanosheets. Both morphologies efficiently degraded organic pollutants because of the extended visible-light-absorption capability of Bi_2_MoO_6_ and excellent separation of charge carriers driven by the photo-induced potential differential of the Bi_2_MoO_6_/TiO_2_ heterojunction [21,22,23,24]. A summary of Bi_2_MoO_6_-based photocatalysts and their photocatalytic performance is illustrated in Table S1 in the Supplementary Materials.

As is widely accepted, enhancement in the surface area could contribute to improving the photocatalytic performance because photodegradation is typically a surface-based process [25]. In this respect, one-dimensional (1D) nanomaterials, including nanowires, nanotubes, and nanorods, have attracted extensive attention because of their large aspect ratio, chemical stability, and unique geometrical morphologies, offering direct pathways for charge transport. On the one hand, nanomaterials with aligned 1D morphologies possess a short diffusion and transport path for holes, along with their radial directions. Simultaneously, their long axes are the preferred channels for electron transfer as well as optical scattering and adsorption [13,14]. Lindquist et al. [26] used Fe_2_O_3_ nanorod arrays as anodes in a photoelectrochemical (PEC) cell to address issues of the PEC system and improve its efficiency. Recent studies also demonstrated superior photocatalytic, photovoltaic, and PEC properties of the aligned 1D nanostructures in addition to their recyclable and reusable characteristics, unlike their bulk or randomly shaped (not aligned) counterparts [27,28,29,30]. On the other hand, nanomaterials with nanosheet morphologies could favor the adsorption of pollutants during photodegradation [31]. However, dispersed Bi_2_MoO_6_/TiO_2_ nanoparticles have downsides owing to their tendency to agglomerate during the reaction and to the difficulty in separating and fully recovering them from the reaction mixture. Few studies have been published on HSs-containing Bi_2_MoO_6_ and 1D TiO_2_ (Bi_2_MoO_6_/TiO_2_ nanobelts, nanotubes, and nanorods) and their photocatalytic performance. Therefore, to further address and explore this topic, a strategy was developed to embed the photocatalytic species on a high surface area material [32,33]. In this context, we developed a simple hydrothermal/solvothermal method to synthesize HSs containing Bi_2_MoO_6_ nanosheets and TiO_2_ nanorod arrays grown on an FTO surface in advance. The crystallinity, structure, morphology, band structure, and optical properties of these HSs were thoroughly analyzed. Our Bi_2_MoO_6_/TiO_2_ HSs exhibited excellent photocatalytic activity in the visible-light region due to the combination of the Bi_2_MoO_6_ light-absorption and charge-separation efficiency of the Bi_2_MoO_6_/TiO_2_ heterojunction.

## 2. Materials and Methods

### 2.1. Materials

All chemicals used in this study were of analytical grade, purchased from Sinopharm Chemical Reagent Co., Tianjing, China and used as received. FTO (SnO_2_:F conducting glass), used as a substrate, was acquired from Kejing Materials Technology Co., Hefei, China. Deionized (DI) water, which was used throughout all experiments, was prepared in our laboratory using the water purifying system RC–K2 (Ruicheng Technology Co., Ltd., Beijing, China).

### 2.2. Preparation of TiO_2_ Nanorod Arrays

TiO_2_ nanorod arrays were grown on an FTO substrate hydrothermally [13,14]. First, a 1:1 (by volume) mixture of hydrochloric acid (HCl, AR, 36.0 ~ 38.0%) and DI water with a specific Ti(OC_4_H_9_)_4_ (AR, 98.0%) content was prepared. Then, the above solution was transferred to a Teflon pot, in which a rectangular piece of FTO, with the conducting layer facing down, was placed against the wall of the Teflon pot. Prior to the synthesis, the FTO was ultrasonicated in DI water, then in acetone, and finally in ethanol. The hydrothermal reaction was performed in a Teflon-lined, stainless-steel autoclave for 6 h at 453 K. The resulting product was TiO_2_ nanorod arrays grown on the FTO pieces.

### 2.3. Synthesis of the Bi_2_MoO_6_/TiO_2_ Composites

Bi_2_MoO_6_/TiO_2_ composites were synthesized solvothermally [34,35,36]. The stepwise synthesis protocol is shown in Figure S1 in the Supplementary Materials. First, Bi(NO_3_)_3_·5H_2_O (AR, 99.0%) and Na_2_MoO_4_·2H_2_O (AR, 99.0%) (at 2:1 mole ratio) were dissolved in a mixture of ethylene glycol (EG, AR, 99.5%) and ethanol (C_2_H_5_OH, AR, 99.7%) (at 1:1 volume ratio) under constant stirring. Then, the resulting clear mixtures were placed into a 50 mL Teflon-lined, stainless-steel autoclave containing an FTO substrate coated with TiO_2_ nanorod arrays. The reaction was conducted at 433 K for 14 h. Under these conditions, several Bi_2_MoO_6_/TiO_2_ composites were prepared with different amounts of Bi_2_MoO_6_ by varying the mass of the raw materials, as shown in Table S2. The corresponding samples were marked as BMT-1, BMT-2, BMT-3, and BMT-4. In addition, pure Bi_2_MoO_6_ nanosheets (without TiO_2_) were also synthesized under the same conditions.

### 2.4. Characterization

The phase structures of the as-prepared products were analyzed by X-ray diffraction (XRD) performed using a D8 Advance (Bruker Corp., Karlsruhe, Germany) instrument equipped with Cu Kα radiation as an X-ray source. The sample morphologies were inspected using scanning electron microscopy (SEM) performed with a JSM-7100F (Hitachi Corp., Tokyo, Japan) instrument. The elemental composition and valence-band potential (*E*_VB_) were obtained using X-ray photoelectron spectroscopy (XPS) performed with an ESCALAB 250 (Thermo Scientific (Shanghai) Corp., China) instrument equipped with Al Kα radiation as an X-ray source. The ultraviolet-visible (UV–Vis) absorption spectra were recorded by a UV2600 (Shimadzu (China) Corp., Shanghai, China) spectrophotometer. The Brunauer–Emmett–Teller (BET) specific surface areas (*S*_BET_) and pore volume of the as-prepared samples were estimated from the nitrogen adsorption−desorption isotherms that were recorded by a nitrogen adsorption apparatus (ASAP 2020, Micromeritics Instruments Corp., Atlanta, GA, USA) at 77 K. Electron spin resonance (ESR) signals of the radicals’ spin were recorded by a E500 spectrometer (Bruker Corp., Karlsruhe, Germany). The 5,5-dimethyl-1-pyrroline N-oxide (DMPO) was selected as a free radical scavenger to capture ·OH and ·O_2_^−^ species. Photoluminescence (PL) emission spectra were measured at room temperature using a FluoTime-300 spectrophotometer (PicoQuant Co., Berlin, Germany) under a 325 nm laser as an excitation source.

### 2.5. Photocatalytic Activity

Methylene blue (MB) was used as a compound representing organic pollutants and other organic dyes. MB is chemically stable and difficult to decompose. The photocatalytic degradation was tested in a MB aqueous solution (1.0 × 10^−5^ mol·L^−1^, 50 mL) using a 1 × 1 cm^2^ FTO substrate containing the Bi_2_MoO_6_/TiO_2_ HSs. A blank sample (without any catalysts) and TiO_2_/FTO nanorod arrays were also used for comparison for the photocatalytic MB degradation experiments. AM 1.5 G simulated solar light (with 100 mW/cm^2^ fluence) was provided by a 300 W Xe PLS-SXE300D (Perfectlight Technology Co., Beijing, China) lamp. Prior to the photocatalytic experiments, the aqueous solutions containing MB and photocatalysts were kept in the dark for 30 min to establish an adsorption/desorption equilibrium. Absorption spectra of MB aqueous solutions, at a wavelength of 664 nm, were collected using a UV–Vis spectrophotometer at specific intervals, which revealed the changes in MB content after irradiation. The dye degradation efficiency was calculated by the formula below [37]:

$$X=\frac{C}{C_{0}}\times 100\%$$

where *C*_0_ and *C* are MB contents at times 0 and *t*, respectively.

### 2.6. Assessment of PEC Performance

The PEC performance of our samples was tested using a CHI660E (Chenhua Instruments Inc., Shanghai, China) electrochemical workstation equipped with a three-electrode (Pt foil as a counter, calomel as a reference, and working electrodes) configuration. In addition, 0.5 M Na_2_SO_4_ served as an electrolyte. The effective area of the working electrodes was 1.5 cm^2^. Electrochemical-impedance-spectroscopy (EIS) was performed with a 5 mV amplitude AC voltage in the 10 Hz–1 MHz range. Mott–Schottky plots of pure TiO_2_ and Bi_2_MoO_6_ were recorded at 1000 Hz. The light source was the same as in the photocatalytic experiment.

## 3. Results and Discussion

### 3.1. X-ray Diffraction (XRD)

**Figure 1:**
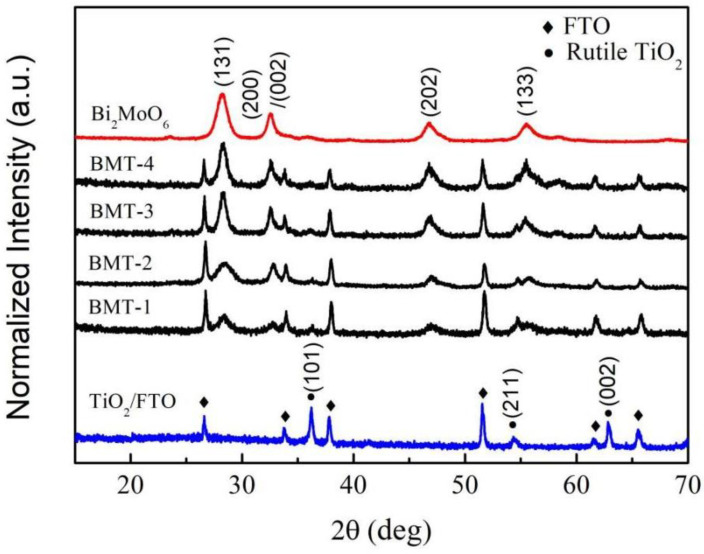
XRD patterns of pristine TiO_2_/FTO, Bi_2_MoO_6_, and BMT-1, BMT-2, BMT-3, and BMT-4 samples.

As shown in Figure 1, synthesized TiO_2_, Bi_2_MoO_6_, and Bi_2_MoO_6_/TiO_2_ composite films possessed good crystallinity according to the XRD results. The XRD pattern revealed diffraction peaks at 36.2°, 54.3°, and 62.8°, attributed to (101), (211), and (002) crystal planes of rutile TiO_2_, respectively (PDF card number 21-1276). The XRD patterns of Bi_2_MoO_6_/TiO_2_ revealed TiO_2_ and FTO peaks, as well as peaks at 28.2°, 32.5°, 46.7°, and 55.5°, that were attributed to (131), (200)/(002), (202), and (133) planes of the orthorhombic koechlinite phase of Bi_2_MoO_6_, according to PDF card number 76-2388. These results confirmed the formation of Bi_2_MoO_6_/TiO_2_ composites. XRD peaks of Bi_2_MoO_6_ observed in the Bi_2_MoO_6_/TiO_2_ spectra became sharper and narrower as the initial Bi_2_MoO_6_ amount was increased, while the XRD peaks of TiO_2_ became less intense. No other changes in the crystalloid structure were observed as the initial materials used were changed.

### 3.2. X-ray Photoelectron Spectroscopy (XPS)

**Figure 2:**
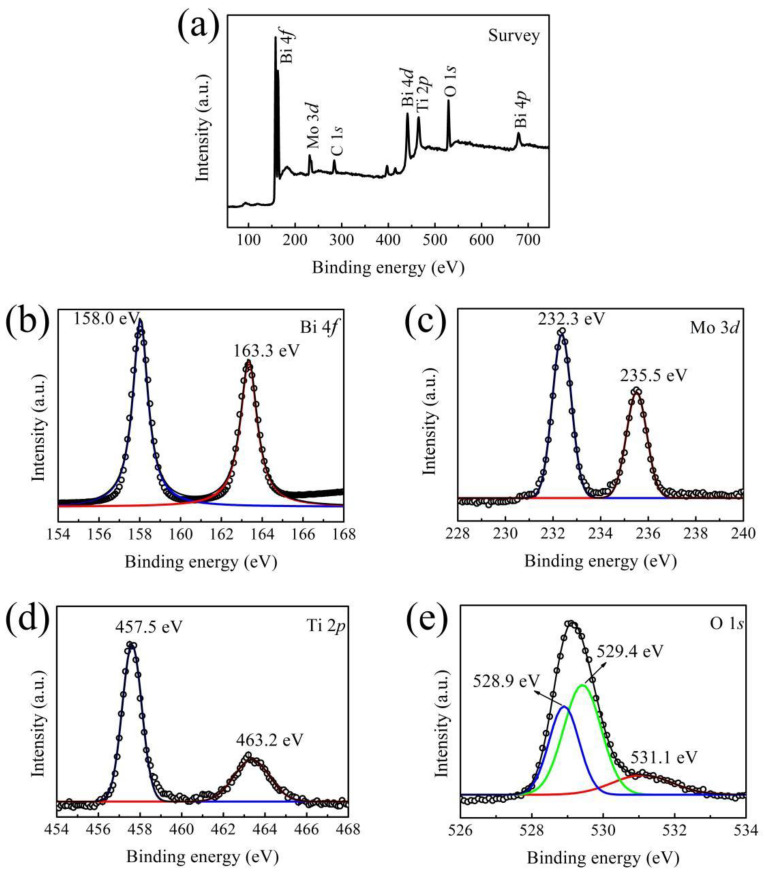
(**a**) XPS fully scanned spectrum of the BMT-3 composite, and high-resolution XPS of (**b**) Bi 4*f*, (**c**) Mo 3*d,* (**d**) Ti 2*p*, and (**e**) O 1*s*. The circle symbol represent the experimental data, the black lines represent the fitting curves, and the colored lines represent the multimodal fitting curves.

XPS of the BMT-3 composites revealed the presence of Bi, Mo, Ti, and O elements (see Figure 2a). Some carbon was also observed due to environmental and instrument contamination. The high-resolution Bi 4*f* XPS spectrum showed two peaks at 158.0 and 163.3 eV (see Figure 2b), which were assigned to the spin-orbit splitting peaks of Bi 4*f*_7/2_ and Bi 4*f*_5/2_, respectively [38]. Thus, Bi in Bi_2_MoO_6_/TiO_2_ existed as Bi^3+^. Two strong peaks at 232.3 and 235.5 eV, corresponding to Mo 3*d*_5/2_ and Mo 3*d*_3/2_ spin-orbit components of Mo^6+^, respectively [39] (see Figure 2c), were observed in the high-resolution Mo 3*d* XPS spectrum. The peaks at 457.5 and 463.2 eV matched the binding energies of Ti 2*p*_3/2_ and Ti 2*p*_1/2_ states, respectively (see Figure 2d). Thus, Ti was present as Ti^4+^ in the Bi_2_MoO_6_/TiO_2_ composites. The fitting of the high-resolution O 1*s* XPS spectrum revealed three peaks at 528.9, 529.4, and 531.1 eV (see Figure 2e), which corresponded to Bi-O, Mo-O, and Ti-O bonds, respectively [22,38]. Thus, these results confirmed the coexistence of Bi_2_MoO_6_ and TiO_2_ in the synthesized HSs.

### 3.3. Morphologies

**Figure 3:**
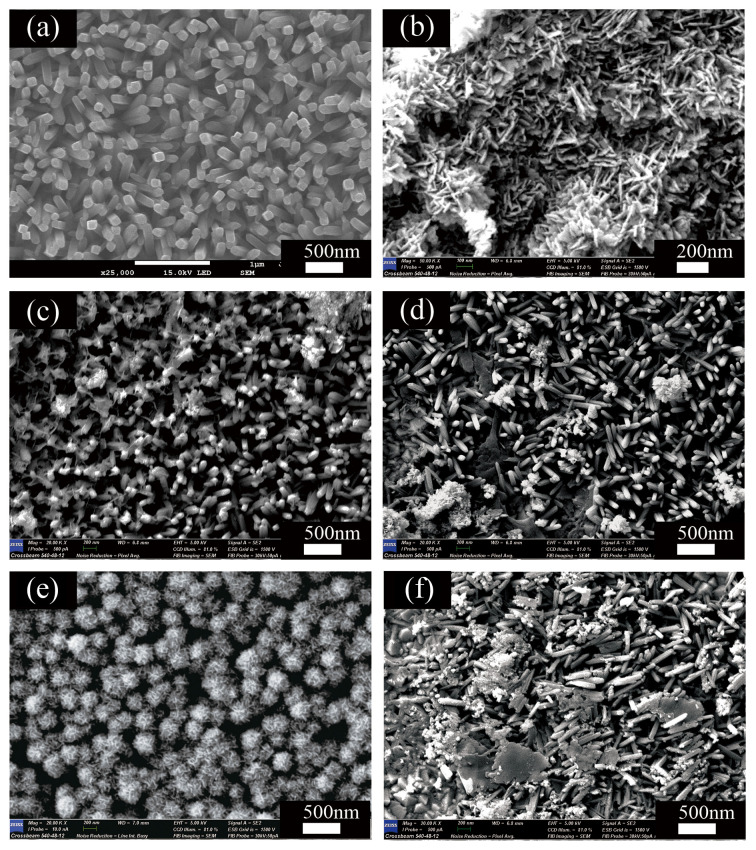
SEM images of (**a**) pristine TiO_2_ nanorod arrays, (**b**) pure Bi_2_MoO_6_ nanosheets, and Bi_2_MoO_6_/TiO_2_ composites denoted as (**c**) BMT-1, (**d**) BMT-2, (**e**) BMT-3, and (**f**) BMT-4.

SEM showed that the diameters of the bare TiO_2_ nanorods were 200 nm (see Figure 3a). Moreover, the nanorods were vertically aligned and uniformly distributed on the FTO substrate. Pure Bi_2_MoO_6_ nanosheets exhibited laminar and irregular, sheet-like morphology (see Figure 3b). SEM of the Bi_2_MoO_6_/TiO_2_ composites showed that some Bi_2_MoO_6_ nanosheets adhered to the TiO_2_ nanorods (see Figure 3c–f). Additionally, as the initial Bi/Mo content was increased, the Bi_2_MoO_6_ nanosheets increased in number and began to aggregate. The sample (BMT-3, shown in Figure 3e) with appropriate initial Bi/Mo content showed the growth of dispersed Bi_2_MoO_6_ nanosheets around TiO_2_ nanorods. Instead, a serious agglomeration of Bi_2_MoO_6_ was observed, as shown in Figure 3f. These findings suggest that the initial concentration of the materials used significantly affected the Bi_2_MoO_6_/TiO_2_ composite morphology.

It is widely known that the photocatalytic activity of catalysts is significantly influenced by the specific surface area and pore volume of photocatalysts due to the presence of surface-reactive sites [40]. The nitrogen adsorption−desorption isotherms of the TiO_2_, Bi_2_MoO_6_, BMT-3, and BMT-4 samples are shown in Figure S2 in the Supplementary Materials. The similar adsorption−desorption curves revealed strong N_2_ adsorption−desorption performance, which indicated the existence of capillary condensation in the large mesopores in the samples. The *S*_BET_ and pore volume of TiO_2_, Bi_2_MoO_6_, BMT-3, and BMT-4 HSs are listed in Table S3. Interestingly, the coupling of TiO_2_ and Bi_2_MoO_6_ significantly increased the *S*_BET_ and pore volume (the maximum value for BMT-3 are 88.2 m^2^/g and 0.18 cm^3^/g, respectively) compared to the pure Bi_2_MoO_6_ (26.0 m^2^/g and 0.05 cm^3^/g, respectively). Combined with the photocatalytic experiment results we can deduce that the high *S*_BET_ and pore volume of BMT-3 played significant roles in enhancing photocatalytic performance.

### 3.4. Optical Properties

**Figure 4:**
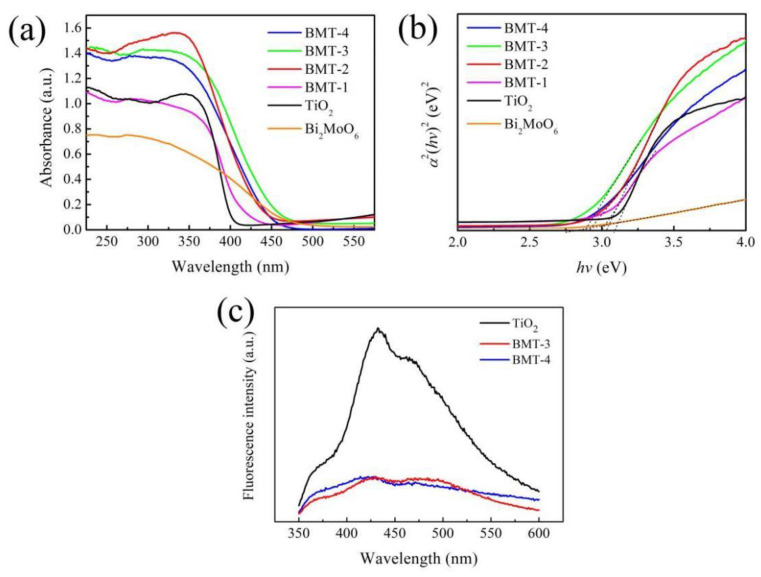
(**a**) UV–Vis absorption spectra: (**b**) Kubelka–Munk plots of pristine TiO_2_ nanorod arrays, pure Bi_2_MoO_6_, and Bi_2_MoO_6_/TiO_2_ composites; and (**c**) PL spectra of TiO_2_ and Bi_2_MoO_6_/TiO_2_ HSs photocatalysts.

The UV–Vis spectra of TiO_2_ nanorod arrays showed that they were adsorbed, as expected, in the ultraviolet region at 400 nm (see Figure 4a), which is mainly accounted for by absorption within the rutile band-gap [41]. The absorption intensity of Bi_2_MoO_6_ was relatively mild and peaked at ~450 nm. Thus, a slight extension to the visible-light region was observed in comparison to TiO_2_. The absorption edges of the Bi_2_MoO_6_/TiO_2_ composites exhibited a certain degree of red-shift, which indicates the extension of the material’s absorption towards visible light. The band-gap energies of the TiO_2_, Bi_2_MoO_6_, and Bi_2_MoO_6_/TiO_2_ HSs were calculated from the Kubelka–Munk function, plotted against the photon energy (see Figure 4b). The band-gap values of TiO_2_ and Bi_2_MoO_6_ were equal to 3.1 and 2.77 eV, respectively. Moreover, the band-gap values of the BMT-1, BMT-2, BMT-3, and BMT-4 composites were 2.88, 2.85, 2.81, and 2.79 eV, respectively. Furthermore, the photogenerated electron-hole pairs were excited due to the extension of absorption into the visible-light region, favoring enhanced PEC and photocatalytic performance.

Photoluminescence (PL) emission spectra have been widely employed to reveal the separation efficiency of the photo-induced electrons and holes in the composite semiconductors [42]. The PL spectra of the as-prepared TiO_2_, BMT-3, and BMT-4 samples in the present study are shown in Figure 4c. The strongest emission intensity peak observed in pure TiO_2_ nanorods corresponded to the band-gap transition. A significant decrease in fluorescence of Bi_2_MoO_6_/TiO_2_ (BMT-3 and BMT-4) was observed, indicating a lower photoelectron-hole recombination in Bi_2_MoO_6_/TiO_2_ than in TiO_2_. Moreover, the similar fluorescence intensity of BMT-3 and BMT-4 indicated similar separation performance of the photo-induced electrons and holes in the BMT-3 and BMT-4 samples. The migration and recombination efficiencies of the photo-induced carriers in all samples were further revealed by EIS characterization.

### 3.5. Photocatalytic Properties

**Figure 5:**
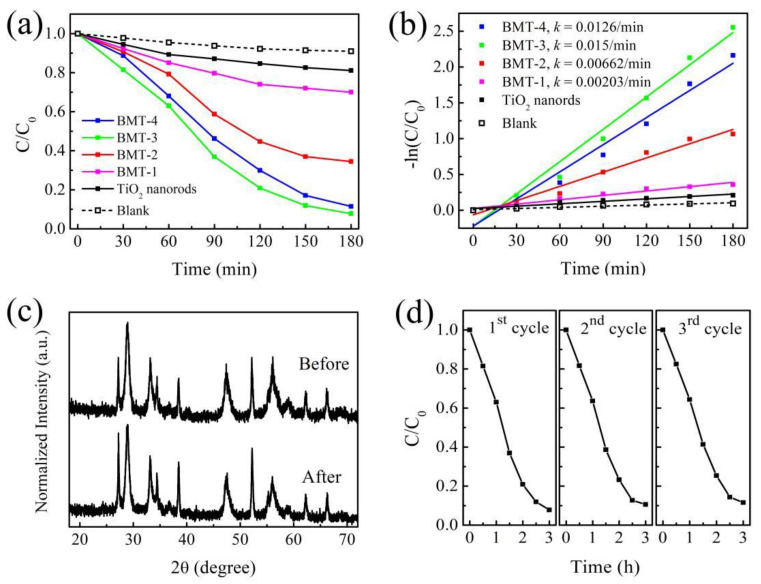
(**a**) Normalized MB concentration versus the irradiation time; (**b**) Kinetic curves of MB photodegradation; (**c**) XRD patterns of original BMT-3 sample and after three recycles photodegradation; (**d**) Repeated photocatalytic activity of BMT-3 under visible-light irradiation for MB degradation.

The relationship between residual MB contents and irradiation time, with and without photocatalyst, is shown in Figure 5a. Figure 5b shows the fitting curves of the kinetics of MB photodegradation. The degradation of MB in the presence of TiO_2_ and Bi_2_MoO_6_/TiO_2_ obeyed the pseudo-first-order kinetics and could be expressed as ln(*C*/*C*_0_) = *k*(*t*−*t_0_*), where *k* is the reaction rate constant [43,44]. The rate constants and the corresponding correlation coefficients (R-Square) of different photocatalysts and the blank sample are given in Table S4, which were calculated by linear fitting −ln(*C*/*C*_0_) to irradiation time (*t*). The values of R-Square are close to 1, which reveal a good correlation to the pseudo-first-order reaction kinetics. MB degradation without catalysts was negligible under visible light, with a *k* value of 0.000538 min^−1^ (see Figure 5b). In contrast, the reaction rate constant of MB decomposition in the presence of TiO_2_ nanorods was 0.00113 min^−1^, which was attributed to the photon-trapping effect caused by the morphology of the TiO_2_ nanostructure [13,14]. However, the photocatalytic performances of the Bi_2_MoO_6_/TiO_2_ HSs were significantly better. Thus, the presence of Bi_2_MoO_6_ in the composite played a significant role. The photocatalytic ability of Bi_2_MoO_6_/TiO_2_ (BMT-4) was lower for samples with higher Bi_2_MoO_6_ content because of the recombination of the photogenerated electron-hole pairs inside the numerous Bi_2_MoO_6_ nanosheets [44]. From the viewpoint of practical application, it is important to evaluate the stability of the as-prepared catalyst. Comparison of the XRD pattern of the Bi_2_MoO_6_/TiO_2_ (BMT-3) before and after the photocatalytic reaction (performed three times with the same catalyst) showed that, even after three cycles, the positions and intensities of the XRD peaks remained almost the same (Figure 5c), which confirmed the excellent stability of the Bi_2_MoO_6_/TiO_2_ composite catalysts. The stable activity was further validated by repeating the photocatalytic degradation processes thrice, as shown in Figure 5d. The three degradation curves showed a similar trend in each running cycle, which indicated that the Bi_2_MoO_6_/TiO_2_ photocatalyst exhibited high and stable activity for degradation.

To investigate the mechanisms underlying the photo-oxidation ability, ESR technology was used to detect active free radicals. The ESR signals of BMT-3 dispersed in the DMPO solution are shown in Figure S3. No ·OH ESR signals were generated in darkness; however, a set of four feature peaks with an intensity ratio of 1:2:2:1 were observed after light illumination (see Figure S3a), attributed to DMPO-·OH adducts. Similarly, no ·O_2_^−^ signals were generated in the dark, and six peaks of DMPO-·O_2_^−^ were observed in the ESR spectra (see Figure S3b) with illumination. These results demonstrated that ·O_2_^−^ and ·OH are both active species, and their synergistic effect significantly promoted the photocatalytic degradation of MB.

### 3.6. PEC Analysis

**Figure 6:**
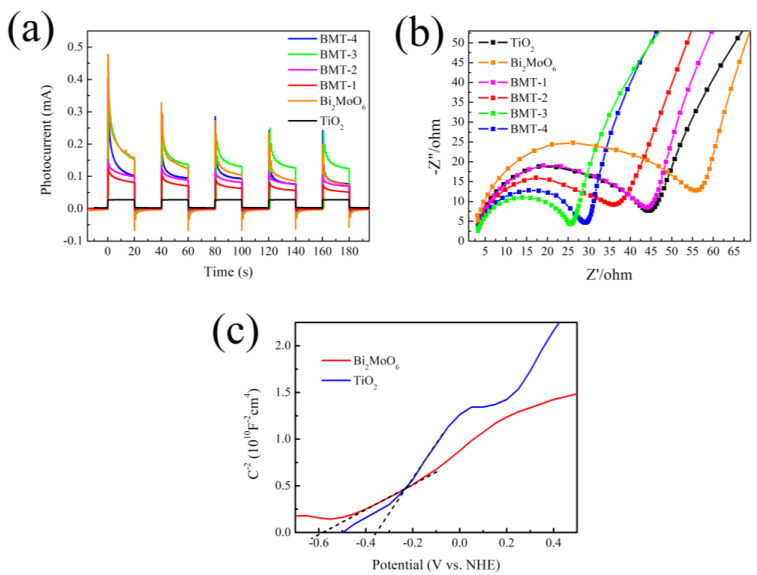
(**a**) The transient photocurrent response; (**b**) Nyquist plots of pristine TiO_2_, Bi_2_MoO_6_, and Bi_2_MoO_6_/TiO_2_; (**c**). Mott–Schottky plots of pristine TiO_2_ and Bi2MoO6.

The PEC performance of the Bi_2_MoO_6_/TiO_2_ HSs was compared to that of pure TiO_2_ nanorod arrays and Bi_2_MoO_6_. For this purpose, we recorded the photocurrent as a function of time (*I*–*t* curves) by alternating exposure to darkness and visible light (see Figure 6a). All samples exhibited similar photocurrent responses. No photocurrent was observed in the dark, which confirmed the absence of any electrochemical processes. A minimal photocurrent response was observed for the TiO_2_ nanorod array, and a significant one for the Bi_2_MoO_6_/TiO_2_ HSs. The BMT-3 sample exhibited optimal stability and reproducibility of the photocurrent response since the Bi_2_MoO_6_/TiO_2_ HSs, and the amount of Bi_2_MoO_6_ was the most favorable out of all the BMT samples. The separation and collection efficiency of interface charges were further studied by EIS (see Figure 6b). Typically, the semicircles in the corresponding Nyquist plots corresponded to Faradic reactions. It has been established that the semicircle radius is negatively correlated with the charge transfer efficiency [45,46]. In our study, the semicircle diameters for the Bi_2_MoO_6_/TiO_2_ HSs were significantly smaller than those obtained for Bi_2_MoO_6_ and TiO_2_. This data confirms the lower interfacial charge-transfer resistance and fast charge-transfer process of our composite Bi_2_MoO_6_/TiO_2_ HSs due to the presence of Bi_2_MoO_6_ and its interfacial interaction with TiO_2_, which enhanced the separation and transfer efficiency of the electron-hole pairs photogenerated in the Bi_2_MoO_6_/TiO_2_ HSs. The heterojunction of the Bi_2_MoO_6_/TiO_2_ interface suppressed the charge recombination, thereby producing more (re)active species, which resulted in a high photocurrent response and photocatalytic activity.

The Mott–Schottky plots of TiO_2_ and Bi_2_MoO_6_ are shown in Figure 6c. The positive slopes of both compounds imply that they are n-type semiconductors. The flat-band potential (*V*_fb_) can be calculated by the Mott–Schottky equation [42]:

$$\frac{1}{C^{2}}=\frac{2}{e\varepsilon \varepsilon_{0}N_{d}}[(V-V_{\mathrm{fb}})-\frac{kT}{e}]$$

where *C* is the capacitance at the interface with the electrolyte, *e* is the electronic charge, $\varepsilon_{0}$ is the vacuum permittivity, ε is the sample dielectric constant, *N*_d_ is the charge-carrier concentration, *V* and *V*_fb_ are the applied and flat-band potentials, *k* is the Boltzmann’s constant, and T is the temperature [47]. The *V*_fb_ could be calculated from the intercept of the 1/*C*^2^ curve (plotted as a *V* function) with the *x*-axis [48]. The *V*_fb_ values for the pure TiO_2_ and Bi_2_MoO_6_ were equal to −0.35 and −0.58 V (vs. a normal hydrogen electrode, NHE), respectively.

### 3.7. Energy Band Alignment and Photocatalytic Mechanism

**Figure 7:**
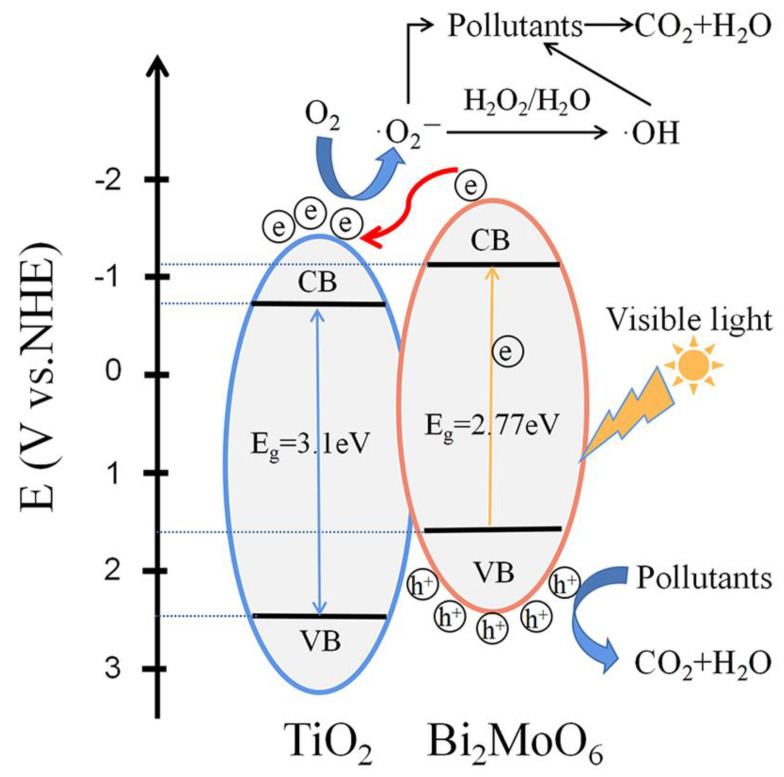
The band structures of the Bi_2_MoO_6_/TiO_2_ HSs and postulated photocatalytic mechanism of dye degradation.

In order to explain the photocatalytic process, the energy band alignment of the Bi_2_MoO_6_/TiO_2_ HSs was investigated. Mott–Schottky plots revealed the flat-band potential of TiO_2_ and Bi_2_MoO_6_ (Figure 6c). A gap between *E*_VB_ and flat-band potential can be inferred from the XPS–valance band (XPS–VB) plots, as shown in Figure S4. Thus, the calculated *E*_VB_ values of TiO_2_ and Bi_2_MoO_6_ were 2.4 and 1.68 V, respectively. Moreover, the conduction band potential (*E*_CB_) of TiO_2_ and Bi_2_MoO_6_ were calculated to be −0.7 and −1.09 V, respectively. Based on these results, the band alignment of the Bi_2_MoO_6_/TiO_2_ was established, as shown in Figure 7. The *E*_CB_ and *E*_VB_ of TiO_2_ were more positive than those of Bi_2_MoO_6_. Accordingly, it is highly likely that Bi_2_MoO_6_/TiO_2_ HSs possess staggered band alignment. Visible-light irradiation excites Bi_2_MoO_6_ molecules, generating electron-hole pairs. At the same time, a large band-gap prevents TiO_2_ molecules from being excited by visible-light irradiation. In this case, the electrons travel from the conduction band of the Bi_2_MoO_6_ to TiO_2_, which suppresses electron-hole pair recombination by the internal field of the Bi_2_MoO_6_/TiO_2_ heterojunction since the *E*_CB_ of Bi_2_MoO_6_ is relatively more negative. The separated electrons can react with O_2_ molecules, forming ·O_2_^−^ radicals since the corresponding redox potential is equal to −0.046 V and more positive than the *E*_CB_ of TiO_2_. Subsequently, the H_2_O molecules are transformed into ·OH radicals after trapping an electron. Both photogenerated holes, together with ·O_2_^−^ and ·OH radicals, can react with MB molecules, damaging their structures. We believe that this process, based on the interfacial charge transfer, is indeed feasible. The reaction mechanism and chemical equations of the above processes are proposed as follows and can be found in the previous literature [23,38,49,50].

$${\mathrm{Bi}}_{2}{\mathrm{MoO}}_{6}+h\nu \to {\mathrm{Bi}}_{2}{\mathrm{MoO}}_{6}\;\left(e^{-}+h^{+}\right)$$

$${\mathrm{Bi}}_{2}{\mathrm{MoO}}_{6}\;\left(e^{-}+h^{+}\right)+{\mathrm{TiO}}_{2}\to {\mathrm{Bi}}_{2}{\mathrm{MoO}}_{6}\;\left(h^{+}\right)+{\mathrm{TiO}}_{2}\;\left(e^{-}\right)$$

$${\mathrm{TiO}}_{2}\;\left(e^{-}\right)+O_{2}\to {\mathrm{TiO}}_{2}+\cdot O_{2}{}^{-}$$

$$\cdot O_{2}{}^{-}+H_{2}O\to \cdot {\mathrm{HO}}_{2}+{\mathrm{OH}}^{-}$$

$$\cdot {\mathrm{HO}}_{2}+H_{2}O\to H_{2}O_{2}+\cdot \mathrm{OH}$$

$$H_{2}O_{2}\to 2\cdot \mathrm{OH}$$

$${\mathrm{Bi}}_{2}{\mathrm{MoO}}_{6}\;\left(h^{+}\right)+\mathrm{MB}\to \mathrm{degraded}\;\mathrm{products}$$

$$\cdot O_{2}{}^{-}+\mathrm{MB}\to \mathrm{degraded}\;\mathrm{products}$$

·OH+MB→degraded products

## 4. Conclusions

In summary, the Bi_2_MoO_6_/TiO_2_ HSs were synthesized through a simple two-step solvothermal process by growing Bi_2_MoO_6_ nanosheets on TiO_2_ nanorod arrays. The Bi_2_MoO_6_/TiO_2_ HSs exhibited enhanced photocatalytic activity for MB degradation under visible-light irradiation, and BMT-3 achieved the highest degradation rate of *k* = 0.015 min^−1^ among all samples. The enhancement was attributed to the heterojunction structures established by the close contact between the Bi_2_MoO_6_ nanosheets and the TiO_2_ nanorods. The results of the UV–Vis absorption spectra show an extended absorption to visible light after coupling Bi_2_MoO_6_ with TiO_2_. The results of N_2_ adsorption−desorption isotherms show a significantly increased *S*_BET_ and pore volume compared to the pure Bi_2_MoO_6_ due to the hierarchical structure of the Bi_2_MoO_6_/TiO_2_ HSs. The results of PL and PEC characterization of the Bi_2_MoO_6_/TiO_2_ HSs reveal improved separation efficiency and enhanced migration rate of photogenerated electron-hole pairs. Thus, the synergy between Bi_2_MoO_6_ and TiO_2_ was identified as the crucial factor leading to the improved photocatalytic performance. Furthermore, the reusability and chemical stability of Bi_2_MoO_6_/TiO_2_ HSs is demonstrated by photocatalytic cycle test. Finally, the mechanism and process of the dye photodegradation were discussed. The h^+^, ·O_2_^−^, and ·OH radicals were validated as being active species that react with MB dye molecules. The results of this work suggest that Bi_2_MoO_6_/TiO_2_ HSs are promising candidate materials for wastewater treatment. Collectively, our current strategy can help in the synthesis and photocatalytic application of other heterostructures in the future.

## Data Availability

All data generated or analyzed during this study are contained within the article and supplementary materials.

## Supplementary Materials

The following supporting information can be downloaded at: https://www.mdpi.com/article/10.3390/nano12030574/s1. Table S1: Bi_2_MoO_6_-based photocatalysts and their photocatalytic performance; Table S2: The abbreviations of products with different amounts of Bi_2_MoO_6_ by varying the mass of raw materials in precursor solution; Table S3: *S*_BET_, Pore Volume, and Mean Pore Diameter of TiO_2_, Bi_2_MoO_6_, BMT-3, and BMT-4 samples; Table S4: Pseudo-first-order rate constants and corresponding R-Square values of different samples; Figure S1: Stepwise synthesis protocol of TiO_2_ nanorod arrays and Bi_2_MoO_6_/TiO_2_ HSs; Figure S2: N2 adsorption−desorption isotherms (a) and the corresponding pore-size distribution curves (b) of TiO_2_, Bi_2_MoO_6_, BMT-3, and BMT-4 samples; Figure S3: The ESR signals of ·OH (a) and ·O_2_^−^ radicals (b) of BMT-3 photocatalysts; Figure S4: XPS valence-band spectra of TiO_2_ and Bi_2_MoO_6_.
