# Supplementary material for: Fabrication of Bi2MoO6 Nanosheets/TiO2 Nanorod Arrays Heterostructures for Enhanced Photocatalytic Performance under Visible-Light Irradiation
Source: Nanomaterials (Basel). 2022 Feb 8;12(3):574. doi: 10.3390/nano12030574 (PMC8840124; doi:10.3390/nano12030574)
Supplement: Supplementary file 1 [file nanomaterials-12-00574-s001.zip › nanomaterials-1565626-supplementary.pdf]

Supplementary Materials

Fabrication of Bi_2MoO_6 Nanosheets/TiO_2 Nanorod Arrays Heterostructures for Enhanced Photocatalytic Performance under Visible-Light Irradiation

Di Zhou [1], Rui Du [1], Zhenglong Hu [2,*], Shu Gao [1], Yafang Tu [1], Yunfei Fu [1], Guang Zheng [1,*] and Youhua Zhou [1]

[1] School of Optoelectronic Materials and Technologies, Jianghan University, Wuhan 430056, China; dizhou@jhun.edu.cn (D.Z.); durui199602@163.com (R.D.); milton_gao@jhun.edu.cn (S.G.); yafangtu@163.com (Y.T.); yffu89@jhun.edu.cn (Y.F.); xinge@whut.edu.cn (Y.Z.)
[2] Laboratory of Low-Dimension Functional Nanostructures and Devices, Hubei University of Science and Technology, Xianning 437100, China
* Correspondence authors: huzhenglong@hbust.edu.cn (Z.H.); mzheng88@foxmail.com (G.Z.)

Table S1. Bi_2MoO_6-based photocatalysts and their photocatalytic performance.

Materials	Degradation rate (%)	Time (min)	Rate constant *k* (min^{-1})	Pollutants	Light sources	Ref.
Bi_2MoO_6 nanosheets	81	150		MB	UV light	[1]
Bi_2MoO_6@TiO_2 nanotubes	21	120		MB	visible light	[1]
Au/Bi_2MoO_6@TiO_2 nanotubes	82	120	0.0203	MB	UV light	[1]
	68	120	0.0081	MB	visible light	[1]
	95	90		BPA	visible light	[1]
Bi_2MoO_6 nanosheets	68.1	80		MO	visible light	[2]
Bi_2MoO_6/TiO_2 nanobelts	90.4	80		MO	visible light	[2]
Bi_2MoO_6/TiO_2 nanobelts	100	40		MO	UV light	[2]
Bi_2MoO_6 nanosheets	29.6	40		MO	UV light	[2]
Bi_2MoO_6/TiO_2 nanobelts	74	60	0.016	X-3B	visible light	[3]
Bi_2MoO_6 microspheres	20	360		MB	visible light	[4]
TiO_2/Bi_2MoO_6	83~95	360		MB	visible light	[4]
Bi_2MoO_6 film	55	240	0.0029	ARS	visible light	[5]
Bi_2MoO_6/TiO_2 bilayer films	85	240	0.0081	ARS	visible light	[5]
TiO_2-Bi_2MoO_6/$Bi_{3.64}Mo_{0.36}O_{6.55}$	93 for RhB, 83 for o-nitrophenol	120		RhB, o-nitrophenol	visible light	[6]
Bi_2MoO_6/TiO_2 microspheres	78~96	300	0.00459~0.00895	phenol	visible light	[7]
	76~94	300	0.00456~0.00812	nitrobenzene	visible light	[7]
Bi_2MoO_6/TiO_2 nanofibers	92	300		RhB	visible light	[8]

	97.3	100	0.03505	MB	visible light	[9]
$Bi/Bi_2MoO_6/TiO_2$ nanotubes	73.21 for MB, 92.98 for RhB	120	0.00739 for MB, 0.02253 for RhB	MB, RhB	solar irradiation	[10]
Bi_2MoO_6/ZIF-8	66.88	100		MB	visible light	[11]
S-doped Bi_2MoO_6	97	60		RhB	visible light	[12]
Bi_2MoO_6/TiO_2 nanorods	95	180	0.015	MB	visible light	This work

Table S2. The abbreviations of products with different amounts of Bi_2MoO_6 by varying the mass of raw materials in precursor solution.

	$Bi(NO_3)_3{\cdot}5H_2O$ (mg)	$Na_2MoO_4{\cdot}2H_2O$ (mg)
BMT-1	20	5
BMT-2	48	12
BMT-3	96	24
BMT-4	144	36

Table S3. S_{BET}, Pore Volume and Mean Pore Diameter of TiO_2, Bi_2MoO_6, BMT-3 and BMT-4 samples.

Samples	S_{BET} (m^2/g)	Pore Volume (cm^3/g)	Mean Pore Diameter (nm)
TiO_2	64.0	0.17	9.7
Bi_2MoO_6	26.0	0.05	3.8
BMT-3	88.2	0.18	4.9
BMT-4	60.7	0.10	3.7

Table S4. Pseudo-first-order rate constants and corresponding R-Square values of different samples.

Samples	Reaction rate constant (*k*) (min^{-1})	R-Square
Blank	0.000538	0.94606
TiO_2 nanorods	0.00113	0.94418
BMT-1	0.00203	0.96246
BMT-2	0.00662	0.96883
BMT-3	0.015	0.95206
BMT-4	0.0126	0.96452

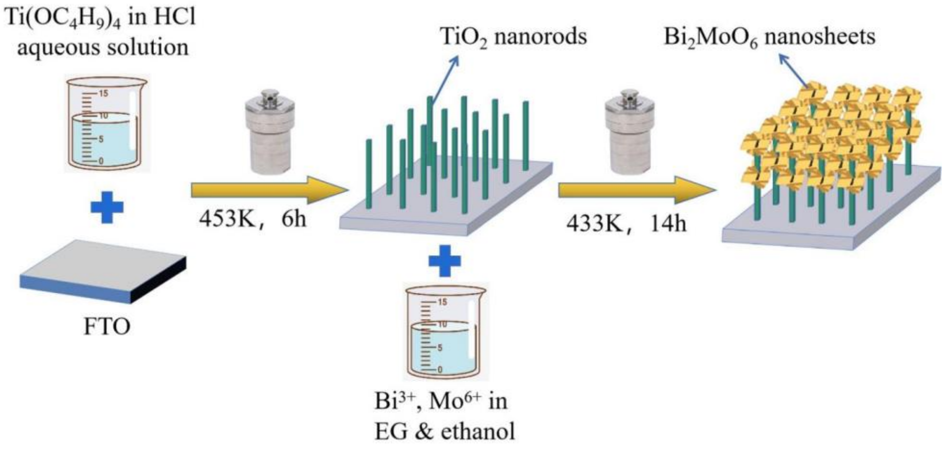

Figure S1. Stepwise synthesis protocol of TiO_2 nanorod arrays and Bi_2MoO_6/TiO_2 HSs.

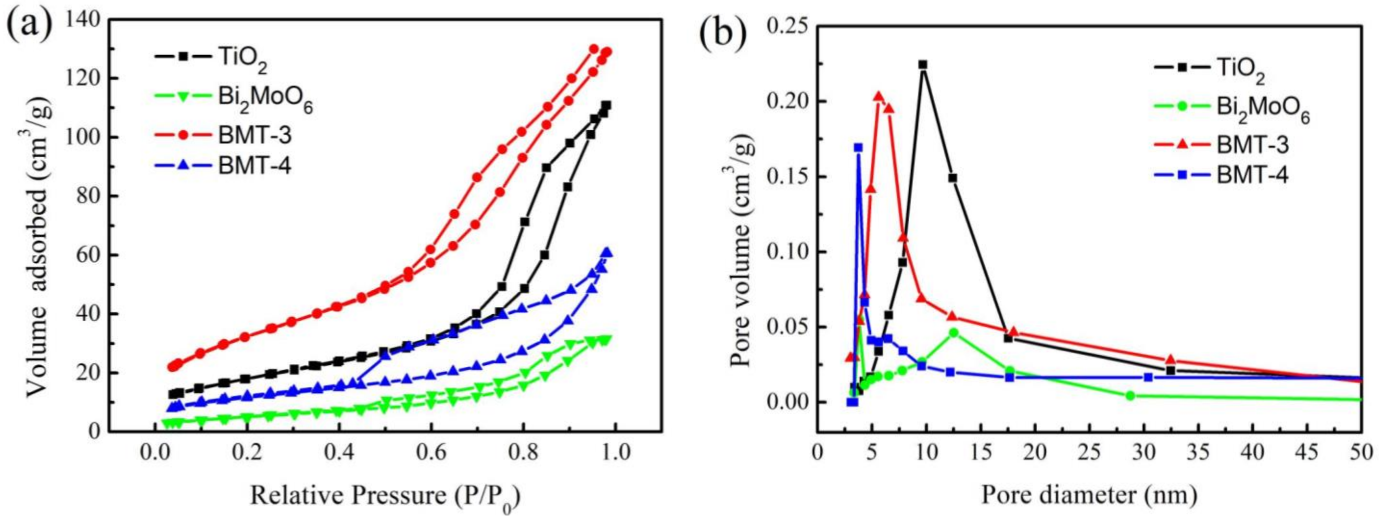

Figure S2. N_2 adsorption−desorption isotherms (a) and the corresponding pore-size distribution curves (b) of TiO_2, Bi_2MoO_6, BMT-3 and BMT-4 samples.

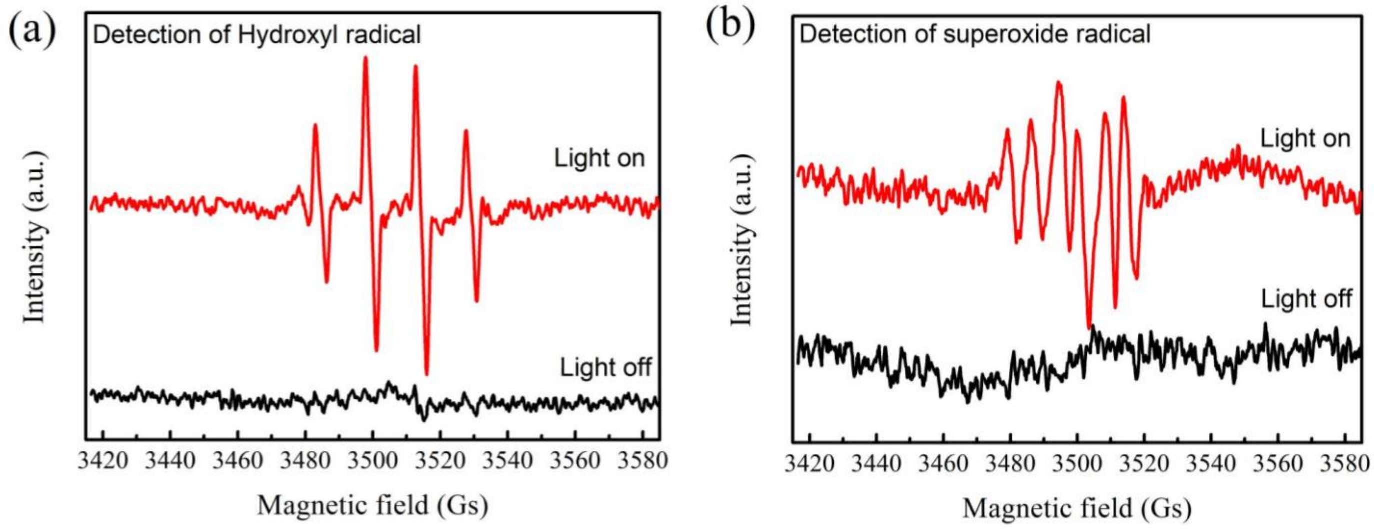

Figure S3. The ESR signals of •OH (a) and $•O_2^-$ radicals (b) of BMT-3 photocatalysts.

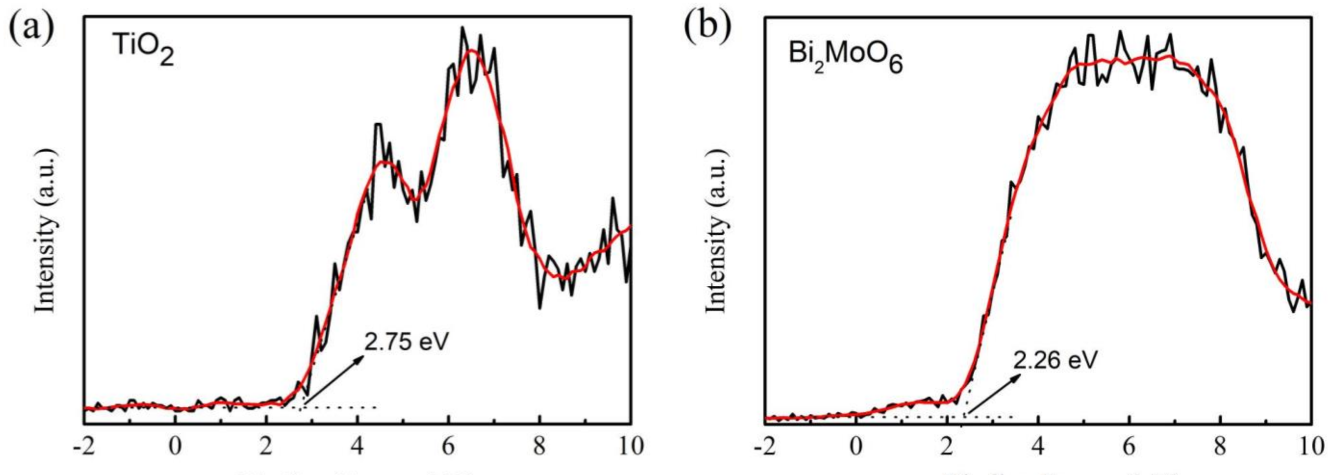

Figure S4. XPS valence band spectra of TiO_2 and Bi_2MoO_6. .
